# Autochthonous Gnathostomiasis, Brazil

**DOI:** 10.3201/eid1812.120367

**Published:** 2012-12

**Authors:** Thiago Jeunon de Sousa Vargas, Sabrina Kahler, Cassio Dib, Marcio Barroso Cavaliere, Maria Auxiliadora Jeunon-Sousa

**Affiliations:** Author affiliations: ID–Investigação em Dermatologia, Rio de Janeiro, Brazil (T. Jeunon de Sousa Vargas, M.A. Jeunon-Sousa);; Hospital Federal de Bonsucesso, Rio de Janeiro (T. Jeunon de Sousa Vargas, S. Kahler, C. Dib);; Colégio Brasileiro de Cirurgiões, Rio de Janeiro (M. Barroso Cavaliere)

**Keywords:** Gnathostomiasis, schistosomiasis, immunoblot, albendazol, ivermectin, autochthonous, parasites, parasitic diseases, nematodes, Brazil

**To the Editor:** Gnathostomiasis is an infestation by nematodes of the genus *Gnathostoma*; the main source of infection is raw freshwater fish. In the past, gnathostomiasis was regarded as restricted to certain Asian and Central American countries, but increase of migratory flux and changes in alimentary habits have contributed to importing cases into areas where the disease is not endemic (1,2). We report a case of autochthonous gnasthostomiasis in Brazil.

A 37-year-old man from Rio de Janeiro sought medical attention in 2005 because of low fever, cough, abdominal tenderness, and pain in the left shoulder. The symptoms started 15 days after a recreational trip to Tocantins, where he practiced sport fishing and ate sashimi-style freshwater raw fish (*Cichla* sp.) that had just been caught. He reported no history of traveling to a gnathostomiasis-endemic area. Initial work-up depicted eosinophilia (43%), and a computed tomographic scan of the chest revealed left pleural effusion. Two weeks later, winding, linear, reddish lesions appeared on his back, which lasted 3 days (Figure, panel A). Serologic testing for *Schistosoma mansoni* was weakly positive. Acute schistosomiasis was diagnosed, and treatment with praziquantel was begun. In 4 weeks, all symptoms faded.

**Figure:**
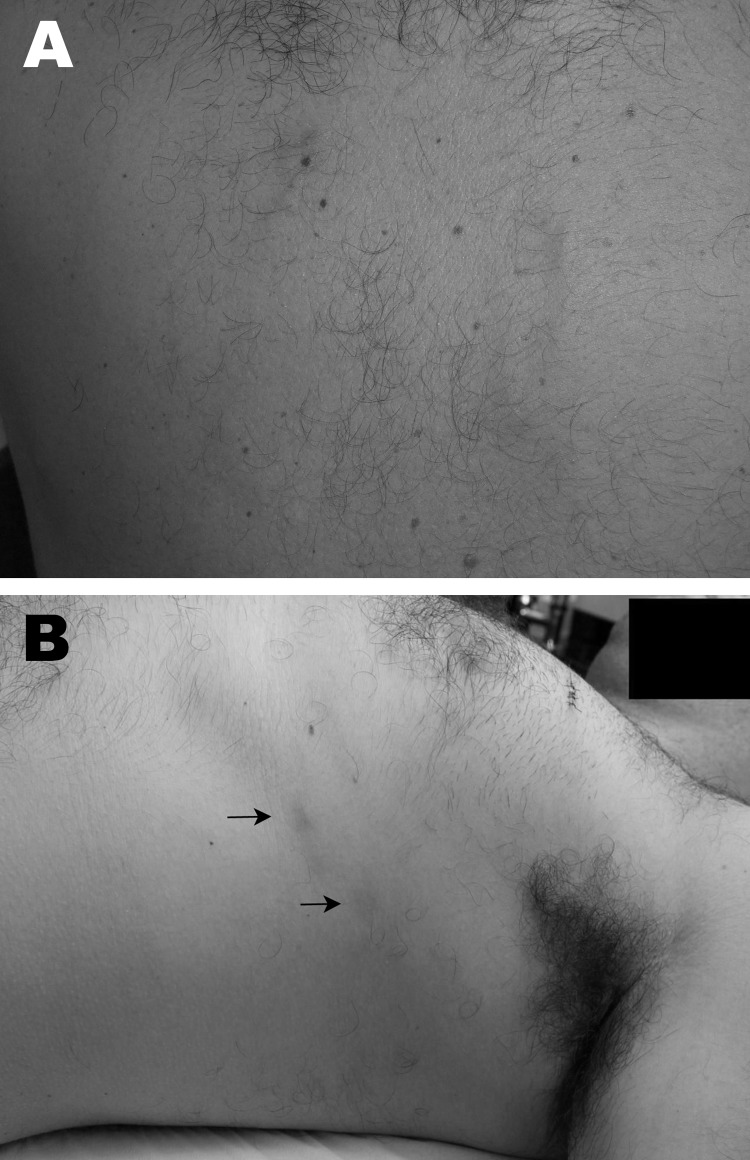
Gnathostomiasis in a 37-year-old man, Brazil. A) Evanescent winding, linear, reddish lesions on the back in 2005. B) Deep migratory reddish nodules (arrows) on the lateral thorax, occurring in 2009 after treatment with albendazol for helmintic prophylaxis.

In 2009, the patient took albendazol for helminthic prophylaxis, and 3 weeks later, deep migratory, swelling, reddish nodules occurred on the thorax; each lesion lasted ≈6 days, and new lesions appeared at intervals of 1–5 days in a somewhat linear array (Figure, panel B). By this time, hemograms displayed eosinophilia of 25%, but a computed tomographic scan of the chest showed no abnormalities. Results of a complete ophthalmologic examination were unremarkable, and a fecal examination was negative for parasites. Gnathostomiasis was highly suspected on the basis of the clinical and epidemiologic findings and results of skin biopsies. Histopathologic examination revealed a dense superficial and deep dermal infiltrate of eosinophils and neutrophils but did not show the parasite. Two samples of plasma were sent to Thailand for immunoblot in search of the diagnostic band (24-kDa antigen) of *Gnathostoma* *spinigerum*, resulting in high titers. Albendazol, 800 mg/day for 21 days, and a single dose of ivermectin, 0.2 mg/kg, were administered and, despite initial improvement, the disease relapsed, requiring a second cycle of the medications. No signs of disease occurred during 2 years of follow-up.

Gnathostomiasis is found mostly in Japan and Thailand. In the Americas, most cases occur in Mexico (3). Gnathostomiasis was previously reported in Brazil, but the patient was infected in Peru (4).

Four species are known to cause disease in humans, and *G. spinigerum* is the most frequent cause. Adult parasites live in the stomach of definitive hosts (dogs, cats, and other fish-eating mammals), and eggs are eliminated in feces. These hatch and release the first-stage larvae in fresh water; larvae are ingested by the first intermediate host, a copepod, and develop into second-stage larvae. Copepods are ingested by the second intermediate hosts (fish, eels, frogs, birds, and reptiles), and larvae mature to the third stage. When eaten by an appropriate definitive host, third-stage larvae evolve to adults and finally reach the stomach of their host.

Third-stage larvae cannot mature in humans and keep migrating in skin, subcutaneous tissue, or other organs. Initial signs and symptoms are fever, anorexia, nausea, vomiting, diarrhea, malaise, urticaria, and epigastric pain. Eosinophilia is frequent. After 2–4 weeks, larvae migrate to skin or subcutaneous tissue, causing winding linear erythematous lesions or migratory swelling nodules. Cutaneous gnathostomiasis is the most common form of disease. The larvae also can migrate to lungs; genitourinary tract; digestive tract; ears; eyes; and rarely, the central nervous system, which may result in death (5). If left untreated, gnathostomiasis may remit and recur several times until death of the larvae ≈12 years after infection.

The rate of detection of larvae in skin biopsy specimens varies from 24% to 34%, and the diagnosis frequently needs confirmation by serologic testing (3). Immunoblot is highly sensitive and specific and is regarded as the most valuable ancillary technique (1,6). The diagnosis in the patient reported here had a 4-year delay, despite investigation in several renowned institutions.

The treatment of choice is albendazol, 800 mg/day for 21 days, but ivermectin, 0.2 mg/kg in a single dose or for 2 subsequent days, is an alternative (7). More than 1 treatment cycle might be required (8). Albendazole promotes outward migration of the larvae to the dermis, and we believe that the low doses used for helminths by the patient reported here might have activated quiescent larvae and triggered new lesions (9).

A high index of suspicion is necessary to diagnose this disease in areas where it is not endemic. Gnathostomiasis must be suspected in a patient who has a history of eating raw freshwater fish, persistent eosinophilia, and larva migrans–like lesions and/or migratory deep nodules in a linear array. History of traveling to gnathostomiasis-endemic areas is not strictly necessary, considering recent reports of gnathostomiasis acquisition in previously unaffected regions (10).
